# Social Distancing, Lockdown and the Wide Use of Mask; A Magic Solution or a Double-Edged Sword for Respiratory Viruses Epidemiology?

**DOI:** 10.3390/vaccines9060595

**Published:** 2021-06-03

**Authors:** Ivan Sanz-Muñoz, Sonia Tamames-Gómez, Javier Castrodeza-Sanz, José María Eiros-Bouza, Raul Ortiz de Lejarazu-Leonardo

**Affiliations:** 1 National Influenza Centre, Edificio Rondilla, Hospital Clínico Universitario de Valladolid, 47009 Valladolid, Spain; TamGomSo@jcyl.es (S.T.-G.); jjcastrodeza@saludcastillayleon.es (J.C.-S.); jmeiros@uva.es (J.M.E.-B.); lejarazu@gmail.com (R.O.d.L.-L.); 2 Dirección General de Salud Pública, Consejería de Sanidad, Junta de Castilla y León, 47001 Valladolid, Spain; 3 Preventive Medicine and Public Health Unit, Hospital Clínico Universitario de Valladolid, 47005 Valladolid, Spain; 4 Microbiology Unit, Hospital Universitario Río Hortega, 47012 Valladolid, Spain

**Keywords:** influenza, respiratory syncytial virus, COVID-19, epidemic, pandemic, respiratory virus

## Abstract

The use of non-pharmaceutical interventions (NPIs), such as social distancing, lockdowns and the massive use of masks, have not only largely prevented the spread of SARS-CoV-2, but also of other respiratory viruses such as influenza or respiratory syncytial virus (RSV). This decrease has been so high that, in most countries, the influenza and RSV epidemic has not occurred. Far from being a beneficial fact, this can be problematic, since the absence of circulation of certain pathogens can lead to a decrease in herd immunity against them. This can promote the rise of more serious, longer-lasting epidemics that start sooner. To alleviate the collateral effects that may occur due to the decrease in circulation of viruses such as influenza, it is necessary to increase the production of influenza vaccines, carry out mass vaccination campaigns and focus on vaccinating the main drivers of this virus, children.

Non-pharmacological interventions (NPIs), such as social distancing, lockdown and the use of masks, have been one of the most effective measures worldwide to reduce the incidence of SARS-CoV-2 during the 2020 pandemic [1]. The mechanism is simple: when the respiratory transmission chain is disrupted by placing physical barriers between the source and vulnerable individuals, transmission is limited and new cases rapidly decline. These factors seem to have greatly restricted the circulation of this new coronavirus, but also had a great impact on the seasonal circulation of influenza in both hemispheres [2,3]. In several southern hemisphere countries on different continents, influenza epidemics were very weak during 2020, and even disappeared in some places [4]. During the 2021 winter in the northern hemisphere, influenza activity almost disappeared, exhibiting an unheard decrease in viral isolates and recording three logs behind the normal findings [5,6]. The same has happened with respiratory syncytial virus (RSV) [7]. Children are the main drivers for both viruses, although, their incidence and clinical relevance are rather different. If we could mimic this behavior for the following seasons, one can conjecture that a great step could be done in the fight against recurring annual respiratory epidemics. However, could this beneficial effect be overshadowed, in the coming years, by more intense epidemics than usual?

Although the mentioned NPIs were also effective against influenza and RSV, other respiratory viruses such as rhinoviruses, adenoviruses, bocaviruses, etc., continue to circulate. In reality, it seems that the viruses most affected by this “viral pandemic stopping” were influenza and RSV, so far. Although the data show that SARS-CoV-2 infections were rarer in children than adults, in Spain the incidence rates of acute-respiratory infections (ARI) clearly showed that the highest ARI rates were in children aged 0–14 years [8]. This appears to be a common global pandemic effect on respiratory virus epidemiology, defined by an interruption of the usual seasonal epidemics of influenza and RSV without altering the epidemiology of other respiratory viruses. This effect was observed for RSV during the 2009 influenza pandemic [9].

Immunity against viral respiratory infections is poorly understood. Protective immunity against certain respiratory viruses has a limited durability over time. Serological protection after exposure to some of them, either through vaccination or naturally, begins to decline even in just a few months. This is well known for influenza, as some authors have determined that serological protection falls between 6–11% each month after the maximum peak of antibodies [10]. Seroprotection after influenza vaccination falls below 60% against both influenza A subtypes (H1 and H3), and influenza B one year beyond immunization, reaching antibody titers similar to those analyzed before vaccination [11]. This sero-evanescence occurs with greater intensity in the elderly than in children. Immune protection against RSV is more complex, and seems to be related to different mechanisms linked to the maturation of the immune system from childhood, reiterative infections throughout life and the quality of the individual development of an immune response against this virus. Some authors have suggested a “variolation” effect of SARS-CoV-2 because mask do not cut 100% of virus transmission, since some droplets and particles can pass through their fabrics [12]. Since this effect has not yet been demonstrated during the COVID-19 pandemic, it is an interesting point that may be studied in the future for other respiratory viruses, such as influenza and RSV.

At the individual level, the absence of circulation of some respiratory viruses does not have to imply an appreciable decrease in protection, since this largely depends on factors intrinsic to each person. However, in society, the accumulation of individuals who are losing this protection and who are not exposed to respiratory viruses for a while, could lead to a pool of susceptible individuals, large enough to cause more severe influenza epidemics in the future, or a greater number of cases than usual for other respiratory viruses. Could NPIs and international travel restrictions impact the “natural” global circulation of the major epidemic respiratory viruses in temperate countries? Is this phenomenon compensated by the circulation of other respiratory viruses or SARS-CoV-2? Will it disappear from geographic areas after massive vaccination against COVID-19? How can this context affect the accuracy and reliability of selection and choice of influenza for the formulation of seasonal vaccines?

Two main points can help to answer the fore-mentioned questions. First, the immune response against the different respiratory viruses is neither the same in duration nor the type of protection conferred by natural infection. The humoral response is only one side of the complex immune response against respiratory viruses. For this reason, the appreciable decrease in circulating antibodies against certain infectious diseases should not be taken as a dogma that indicates that protection decreases in unison. Despite observing this decrease through laboratory tests, the ability to fight certain infections is contained both in memory B lymphocytes (humoral memory) and in TCD4 and TCD8 lymphocytes (cellular memory). However, given that the duration of this memory is variable between different microorganisms and depends, in many cases, on each person and their clinical condition, the lasting lack of exposure could be harmful. On the other hand, if a virus like human influenza does not circulate, the antigenic drift would be lower than normal or almost disappear. This can redound in a lower immune scape of the virus.

Second, the epidemiologies of respiratory viruses share similarities, but also show notable differences between them. For example, after a low intensity influenza epidemic due to a mild winter, 72% of the next epidemics tend to be more intense and more severe than average, starting 11 days sooner and causing 40% more cases on average [13]. This phenomenon happens because, during warm winters, the rate of transmission of influenza is lower than usual and that implies the natural immunization of a smaller number of people. Therefore, it creates a greater pool of susceptible individuals during the next season due to a drop in herd immunity. The current situation, in which a lower incidence of influenza and RSV is occurring in a forced way by NPIs, could mimic the production of more intense epidemics after mild winters (Figure 1). As an example, early reports from New Zealand, Australia and recently France, showed an “out of season” presentation of RSV in children, with unusual epidemic features [14].

Current NPI measures have been tremendously effective in limiting the COVID-19 pandemic and also the spread of other respiratory viruses such as influenza [15]. However, it is necessary to take into account that the lack of exposure to certain pathogens can have unpredictable consequences in the future, and can prolong the effects of the pandemic long after its end. It is mandatory to resize the health systems through profound reforms, adapting them to situations of increased demand and providing surveillance systems with greater capacity to predict these types of situations. One of the most important measures to take to face the decrease in immunity is to develop an “influenza enhanced vaccination strategy” during the 2021–2022 vaccine campaign, as a way to artificially reduce the percentage of susceptible individuals to face the next influenza epidemic, and to limit the damage that a potential higher and earlier circulation could imply. This may involve the increase of influenza vaccine production, the development and schedule of massive vaccination programs like for COVID-19 and also awareness campaigns for populations, above all to increase children vaccination.

In the coming months we will have to look closely at what is happening with the main epidemics of respiratory viruses in the countries of the southern hemisphere, and take note of it. Whether the effect of wearing mask, lockdowns, social distancing measures and international travel restrictions become a double-edged sword solution for influenza and RSV epidemics is a question that deserves future research and observational studies.

## Figures and Tables

**Figure 1:**
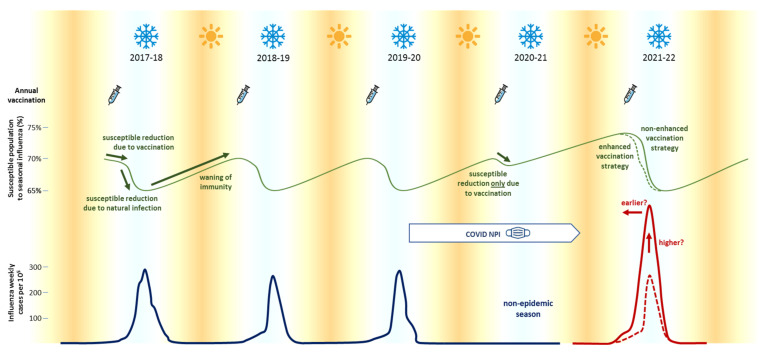
Seasonal influenza epidemics occurred yearly during the winter at the tempered weather latitudes (blue line). Susceptible population (solid green line) declines in autumn due to vaccination campaigns, and later, in winter, due to natural infection. Non-pharmacological interventions (NPIs) to prevent COVID-19 have reduced the incidence of influenza epidemics during the 2020–2021 season. Theoretically, once NPI measures are relaxed, the next influenza epidemic could occur sooner and reach higher incidence (solid red line) due to the higher percentage of susceptible population. In order to ensure that the next influenza epidemic occurs within the usual limits (dotted red line), it would be necessary to enhance the vaccination strategy for reducing the susceptible population at least to the levels otherwise reached through natural infection (dotted green line), or to adopt measures to reduce the intensity of infection, such as children vaccination strategies.

## Data Availability

Data sharing not applicable.
